# Cases of alcohol poisoning involving in-patient treatment

**DOI:** 10.17886/RKI-GBE-2016-026

**Published:** 2016-09-28

**Authors:** Martina Rabenberg, Rommel Alexander, Saß Anke-Christine

**Affiliations:** Robert Koch Institute, Department for Epidemiology and Health Monitoring, Berlin, Germany

**Keywords:** ALCOHOL POISONING, IN-PATIENT TREATMENT, ADOLESCENTS, ADULTS, GERMANY

## Abstract

Alcohol poisonings represent the direct consequences of excess alcohol consumption. In 2014, in Germany 115,967 persons aged 10 to 79 years were treated as in-patients with the diagnosis “acute alcohol intoxication”. In almost all age groups, male persons are affected significantly more frequently than female persons. In the past 14 years, the number of alcohol poisonings involving in-patient treatment amongst children, adolescents and adults has more than doubled. The need to prevent excessive alcohol consumption remains a key objective. Preventive measures should begin while children are still at an early age and in adolescence.

## Introduction

Alcohol consumption is widespread in Germany [1-4]. The individual consequences of high-risk, abusive or addictive consumption include damage to internal organs, cancer, cardio-vascular and gastrointestinal disorders, mental and neurological afflictions and higher mortality [5-8]. Moreover, direct individual consequences of alcohol consumption include increased danger of accidents and injuries as well as acute alcohol poisoning (alcohol intoxication). The extent of intoxication can vary significantly in individual cases and depends on the level of alcohol concentration in the blood. Consequences of intoxication can therefore cover a wide spectrum. These include an upset sense of balance, speech impediments, delayed reaction times, reduced pain perception, nausea and vomiting, unconsciousness and, in extreme cases, death.

Prevention of alcohol-related effects represents a key element of numerous public health strategies. For instance, the “Global Action Plan for the Prevention and Control of Non-Communicable Diseases 2013-2020” of the World Health Organization (WHO) calls for a relative reduction of high-risk alcohol consumption by 10% between the years 2010 and 2025 [9]. In Germany, a reduction in alcohol consumption and the consequences thereof has become part of the national health targets [10].

To be able to assess the level of target achievement, continual monitoring of central indicators is indispensable. Accordingly, in its action plan the WHO proposes a measurement of per-capita consumption of pure alcohol, the prevalence of severe binge drinking and alcohol-related morbidity and mortality as core indicators and recommends that this list be extended in a national context. In the present edition of Public Health Monitoring, information on high-risk alcohol consumption and on per-capita consumption is prepared as part of a focus (link to focus). In addition, there are fact sheets on traffic accidents under the influence of alcohol and on alcohol-related mortality. The present fact sheet augments this information to include in-patient treatment on account of acute alcohol poisoning.

## Indicator

Anyone treated for acute alcohol intoxication as an in-patient is documented in the Hospital Diagnosis Statistics of the German Federal Statistical Office. The Hospital Diagnosis Statistics are an annual total survey that is carried out by the German Federal Statistical Office, in which data on patients under full-time institutional care at all German hospitals are recorded, including their gender, age and main diagnosis [11]. The diagnosis is coded according to the diagnosis codes of the 10th Revision of the “International Statistical Classification of Diseases and Related Health Problems” (ICD-10). For the evaluations of the fact sheet, reference was made to the ICD-10 code F10.0 “Mental and behavioural disorders due to use of alcohol: acute intoxication” [12].

As an indicator, the absolute number of treatment cases in 2014 and in the course of time from the year 2000 to 2014 is evaluated by gender and age. In addition, the relevant rates per 100,000 inhabitants are analysed. The calculations include persons aged 10 to 79 years.

## Classification of findings

In 2014, a total of 115,967 persons aged 10 to 79 years were treated as in-patients with the main diagnosis “acute alcohol intoxication” (Table 1). In the age group of 10 to 19-year-olds, amongst girls 9,254 cases (245 cases per 100,000 inhabitants) and amongst boys 12,975 cases (325 cases per 100,000 inhabitants) were registered. In the category of women aged 20 to 79 years, 25,260 cases (81 cases per 100,000 inhabitants) were recorded in the statistics; amongst men of the same age, there were 68,478 cases (224 cases per 100,000 inhabitants).

**Fig. 1:**
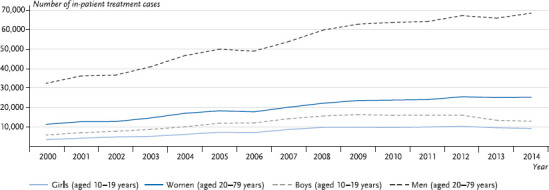
Trend of in-patient treatment for alcohol intoxications over time (ICD-10 Code F10.0) by gender and age Source: Hospital Diagnosis Statistics [12]

Male persons were treated significantly more frequently as in-patients than female persons in almost all age groups. Only in the category of 10 to 14-year-olds were more girls involved than boys. In the course of time, it is evident that the frequency of acute alcohol intoxications involving in-patient treatment increased significantly from the year 2000 to 2014 (Fig. 1). Amongst girls aged 10 to 19 years, it was up by 162%, compared with +120% amongst boys of the same age. Since 2012, however, there has been evidence of a slight downward trend in the number of cases treated amongst 10 to 19-year-olds of both genders. Amongst women aged 20 to 79 years, in-patient treatment for alcohol intoxications was up by a total of 121% from the year 2000 to 2014, compared with 112% amongst men of the same age. In both genders, there has been an almost constant upward trend in the frequency of treatment cases since the beginning of the new millennium. Regional differences have also been observed. For instance, in 2014 the highest treatment rates were recorded in the Saarland region, in Saxony-Anhalt and Rhineland-Palatinate with 237, 210 and 206 cases per 100,000 inhabitants, respectively; in Hamburg, Berlin and Brandenburg, the lowest rates were recorded with 68, 85 and 124 cases per 100,000 inhabitants, respectively [12].

Alcohol poisonings mostly occur due to episodically excessive alcohol consumption, also referred to as binge drinking [1, 13]. This is defined as drinking 60 g or more of pure alcohol on a single occasion. In Germany, this corresponds to a volume of at least five standard drinks at 12g of alcohol each [1]. The frequency of binge drinking (prevalence) in Germany is determined via various epidemiological surveys. A comparison of survey data with the data of the Hospital Diagnostic Statistics shows that the trends of a 30-day prevalence of binge drinking do not necessarily reflect the trends of in-patient treatment of acute alcohol intoxications. For instance, evaluations of the representative alcohol survey 2014 show that the 30-day prevalence of binge drinking amongst children and adolescents has declined considerably. From 2007 to 2012, the proportion of 12 to 17-year-olds in these figures halved, from 25.5% to 12.9% [14]. Amongst adults, in contrast, there is a slightly differentiated outcome. While the prevalence amongst 18 to 59-year-olds stagnated from the year 2000 to 2012, in the age group of 18 to 24-year-olds it increased significantly from 37.0% to 42.3%, and in the age group of 25 to 39-year-olds significantly from 28.6% to 31.9% [15]. Various reasons can be presented to explain these discrepancies. For one thing, it is possible that the frequency of regular binge drinking as such has declined, whereas selective excessive drinking giving rise to alcohol intoxications in need of treatment has risen. For another, methodological differences may play a part. As the Hospital Diagnosis Statistics are not evaluated in terms of persons but in a case-based manner, it is not possible to draw any conclusions regarding the number of times a certain patient has been taken to hospital, for instance. Multiple treatments of a single person for acute alcohol intoxication are therefore included as independent cases in the statistics. It cannot be ruled out either that changed coding habits of medical practitioners have contributed to the increase in the number of cases in recent years. Moreover, experts assume that part of the increase may be attributable to an increased perception of excessive alcohol abuse in public places and to an elevated readiness amongst the population to make emergency calls [16-18].

Analyses like the present one indicate that it remains necessary to prevent excessive alcohol consumption. In this context, a policy mix of behaviour-related and situation-based preventive measures is recommended [19, 20]. In Germany, for instance, this is used as part of the national health target “Reduce alcohol consumption”, within the scope of the nationwide alcohol prevention project „HaLT – Hart am LimiT“ (www.halt-projekt.de) as well as in various campaigns launched by the Federal Centre for Health Information (Bundeszentrale für gesundheitliche Aufklärung) [10, 21].

The implementation of measures should begin while children are still at a young age and in adolescence as the attitudes and behavioural patterns in relation to alcohol predominantly develop at an earlier age and generally continue to prevail until adulthood [3].

## Key statements

- In 2014, a total of 115,967 persons aged 10 to 79 years were treated as in-patients with the main diagnosis “acute alcohol intoxication”.
- Male persons were treated significantly more frequently than female persons in almost all age groups. Only in the category of 10 to 14-year-olds were more girls involved than boys.
- From the year 2000 to 2014, the number of alcohol poisonings requiring in-patient treatment more than doubled in all age groups; however, a slight decline has been recorded since 2012 amongst 10 to 19-year-olds.

## Figures and Tables

**Table 1:** In-patient treatment cases of alcohol intoxications (ICD-10 Code F10.0) by gender and age Source: Hospital Diagnosis Statistics [12]

	Girls and women		Boys and men	
	Number of cases	Number of cases per 100,000 inhabitants	Number of cases	Number of cases per 100,000 inhabitants
**Age**				
10 – 14 years	1,767	97	1,173	61
15 – 19 years	7,487	380	11,802	566
20 – 29 years	5,967	126	13,455	270
30 – 39 years	4,121	85	11,682	237
40 – 49 years	5,814	97	16,310	266
50 – 59 years	5,492	88	16,917	269
60 – 69 years	2,465	52	7,260	165
70 – 79 years	1,401	30	2,854	74
**Total**	34,514	99	81,453	235
